# Diaphragm ultrasonography as a monitor in assessing antagonistic effect of sugammadex on rocuronium in patients with Child-Pugh grades A and B

**DOI:** 10.3389/fmed.2024.1370021

**Published:** 2024-04-05

**Authors:** Yan Sun, Shujun Sun, Rui Chen, Jiwei Shen, Xiangdong Chen, Yun Lin, Shanglong Yao

**Affiliations:** ^1^Department of Anesthesiology, Union Hospital, Tongji Medical College, Huazhong University of Science and Technology, Wuhan, China; ^2^Institute of Anesthesia and Critical Care Medicine, Union Hospital, Tongji Medical College, Huazhong University of Science and Technology, Wuhan, China; ^3^Key Laboratory of Anesthesiology and Resuscitation (Huazhong University of Science and Technology), Ministry of Education, Wuhan, China

**Keywords:** Child-Pugh grades, diaphragm ultrasonography, neuromuscular monitoring, postoperative residual curarization, rocuronium, sugammadex

## Abstract

**Background:**

Although diaphragm ultrasound can be used for detecting residual neuromuscular blockade post-surgery, there exists notable dearth in contemporary research exploring the correlation between preoperative Child-Pugh classification and the effectiveness of sugammadex in reversing rocuronium-induced blockade as evaluated by diaphragmatic ultrasonography.

**Methods:**

This was a prospective, double-blind, non-randomized controlled clinical trial conducted on patients scheduled for laparoscopic liver resection surgery. The participants were categorized into two groups, A and B, based on their preoperative Child-Pugh classification. Prior to anesthesia induction, baseline diaphragm thickness was evaluated using ultrasonography. Throughout the surgical procedure, a deep neuromuscular blockade was maintained with rocuronium. Post-surgery, sugammadex (2 mg/kg) was intravenously administered to patients in both groups upon reaching a train-of-four ratio of 0.2. Diaphragm thickness was assessed at 0, 10, and 30 min, as well as 2 h after extubation, to analyze thickening fractioning (TF) and thickness recovery fractioning (TRF).

**Results:**

No significant differences in TF or TRF were observed between the two groups at 0, 10, and 30 min, as well as 2 h after extubation. Furthermore, there were no significant variances in hemodynamic stability following sugammadex administration. However, patients in the Child-Pugh B group experienced a significantly prolonged time from sugammadex administration to tracheal extubation (19 ± 8.0 min vs. 11 ± 6.1 min) and an extended post-anesthesia care unit stay (123 ± 28.3 min vs. 103 ± 26.0 min) compared to those in the Child-Pugh A group.

**Conclusion:**

The preoperative Child-Pugh grades may not exhibit a significant association with the reversal effect of sugammadex on rocuronium, as evaluated through diaphragmatic ultrasonography.

**Clinical trial registration:**

Registered in the ClinicalTrials.gov (NCT05028088) on July 18, 2021.

## Background

Surgery is the primary treatment modality for patients with hepatocellular carcinoma (HCC), a population frequently characterized by varying degrees of liver dysfunction (1). Laparoscopic radical resection, known for its minimally invasive nature, has emerged as a preferred approach for many HCC patients. The application of deep neuromuscular blockade during laparoscopic procedures has been identified as an effective strategy to reduce intra-abdominal pressure and enhance patient outcomes (2, 3). Nevertheless, concerns persist regarding the risk of severe complications stemming from postoperative residual curarisation (PROC).

Rocuronium, a commonly used non-depolarizing muscle relaxant in general anesthesia, primarily undergoes hepatic metabolism for clearance. Liver impairment in patients results in an increased distribution volume and prolonged elimination half-life of rocuronium (4, 5). Sugammadex, a potent and specific antagonist for aminosteroid muscle relaxants, acts by selectively encapsulating rocuronium to form a one-to-one complex. This complex is rapidly excreted via the kidneys, facilitating a prompt reversal of neuromuscular blockade at varying depths within minutes and with minimal adverse effects (6, 7). When managing PORC, anesthesiologists must focus on two key aspects: the correct administration of muscle relaxant antagonists and the proper utilization of neuromuscular monitoring techniques. While a train-of-four ratio (TOFR) < 0.9 as detected by objective neuromuscular monitoring serves as the current gold standard for diagnosing PORC in clinical settings (8, 9), the routine use of neuromuscular monitoring encounters technical challenges (10).

The diaphragm, being a vital respiratory muscle, exhibits significant resistance to neuromuscular blocking agents and is typically the first muscle to recover from relaxation (11). Research has demonstrated the feasibility of employing ultrasound to monitor diaphragm function (12, 13). Moreover, Cappellini et al. conducted a subsequent investigation on diaphragm ultrasonography, proposing that a thickening fractioning of 0.36 or lower could define PORC (14).

In this study, we employed ultrasonography to evaluate diaphragm thickness in surgical patients categorized as Child-Pugh grades A and B. Our aim was to investigate the association between different Child-Pugh grades and the reversal effect of sugammadex on rocuronium.

## Methods

### Study design

**Figure 1:**
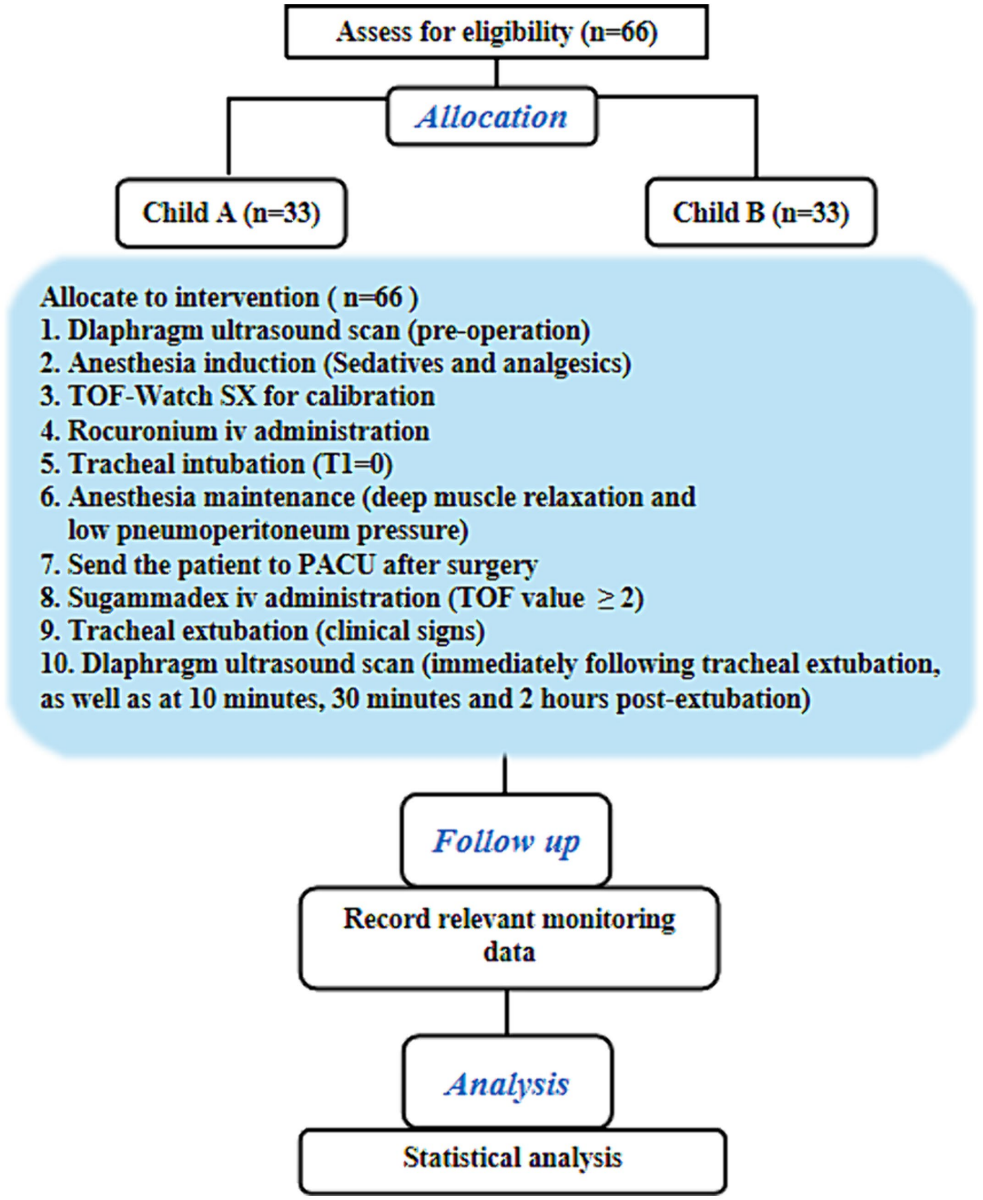
The study flow diagram. PACU, postanesthesia care unit; TOF, train of four.

The study was conducted at the Department of Anesthesiology in Union Hospital of Tongji Medical College, Huazhong University of Science and Technology, Wuhan, China. It was registered in ClinicalTrials.gov (NCT05028088) on 18/07/2021. In compliance with ethical guidelines, written informed consent was obtained from all participants. A total of 66 participants diagnosed with hepatocellular carcinoma, aged 18 to 65 years, and with a body mass index (BMI) ranging from 18.5 to 24.9 kg/m2, were enrolled in this study. The participants included an equal number of Child-Pugh A and B cases scheduled for laparoscopic radical resection. Exclusion criteria included hypersensitivity to rocuronium and sugammadex, the presence of central or peripheral nervous system disorders (e.g., Parkinson’s disease or peripheral neuropathy), neuromuscular disorders (such as multiple sclerosis, myasthenia gravis, and atrophic myotonia), diaphragmatic dysfunction, or the presence of pneumothorax, pleural effusion, or mediastinal pneumatosis. Pregnancy or lactation were also among the exclusion criteria. Patients were categorized into groups A and B based on the Child-Pugh scoring system (Table 1) (15). Demographic data, including age, gender, BMI, and medical history, were collected from medical records or patient interviews. Please refer to Figure 1 for the study flow chart.

**Table 1:** Patients’ characteristics.

	Child-Pugh A (*n* = 33)	Child-Pugh B (*n* = 33)
Baseline and surgical characteristics		
Age (Mean, SD)	56 ± 11.9	55 ± 13.0
Gender (males; n,%)	15(0.5)	16(0.5)
BMI* (kg/㎡; Mean, SD)	23.1 ± 2.7	22.5 ± 3.4
Surgery duration (min; Mean, SD)	202 ± 108.4	249 ± 118.3
Anesthesia duration (min; Mean, SD)	258 ± 116.0	298 ± 120.4
Hypertension (n,%)	11(0.3)	13(0.4)
Cardiopathy (n,%)	6(0.2)	4(0.1)
Diabetes (n,%)	4(0.1)	3(0.1)
Pulmonary disease (n,%)	7(0.2)	4(0.1)
Intraoperative sufentanil dosage (ug; Mean, SD)	25 ± 4.2	25 ± 4.6
Intraoperative remifentanil dosage (mg; Mean, SD)	3.4 ± 1.5	3.9 ± 1.7
Intraoperative propofol dosage (mg; Mean, SD)	1801 ± 655.6	1948 ± 618.5
intraoperative rocuronium dosage (mg; Mean, SD)	154 ± 45.1	181 ± 71.1
Sugammadex dosage (mg; Mean, SD)	124 ± 19.5	124 ± 25.2
The liver function test		
Encephalopathy	1.0 ± 0.0	1.0 ± 0.0
Ascites	1.1 ± 0.2	1.4 ± 0.6
Bilirubin	1.0 ± 0.0	2.5 ± 0.8
Albumin	1.1 ± 0.3	1.8 ± 0.4
Prothrombin Time	1.0 ± 0.0	1.0 ± 0.0
Score	5.2 ± 0.4	7.6 ± 0.7

*BMI, body mass index.

### Neuromuscular monitoring

Neuromuscular blockade in supine patients was assessed using acceleromyography (TOF-Watch® SX, Organon Ireland Ltd., a subsidiary of Merck & Co., Inc., Swords, Co. Dublin, Ireland) at the adductor pollicis muscle. The upper forearms were positioned with a supinated palm and secured onto an arm board. The skin surface was prepared by abrading and cleaning with an alcohol swab, followed by air drying. TOF-Watch electrodes were placed at specific locations: the distal electrode at the intersection between the radial border of the flexor carpi ulnaris muscle and the proximal margin of the wrist curve, and the proximal electrode positioned 2–3 cm away from it. Additionally, two surface electrodes were attached to the forearm where the ulnar nerve is located. The transducer position was secured using a hand adapter, with a temperature sensor affixed to the distal end of the forearm. To maintain a minimum arm temperature of 32°C, the patient was draped with warming blankets. Calibration of the TOF Watch was performed once the BIS value dropped below 60 for each patient before administering muscle relaxants during anesthesia induction. Throughout the surgery, TOF stimuli were repeated every 15 s. Deep neuromuscular blockade was defined as no response to TOF stimulation but a response to post-tetanic-count (PTC) stimulation (1 to 2) (16).

### Diaphragm thickness measurements

**Figure 2:**
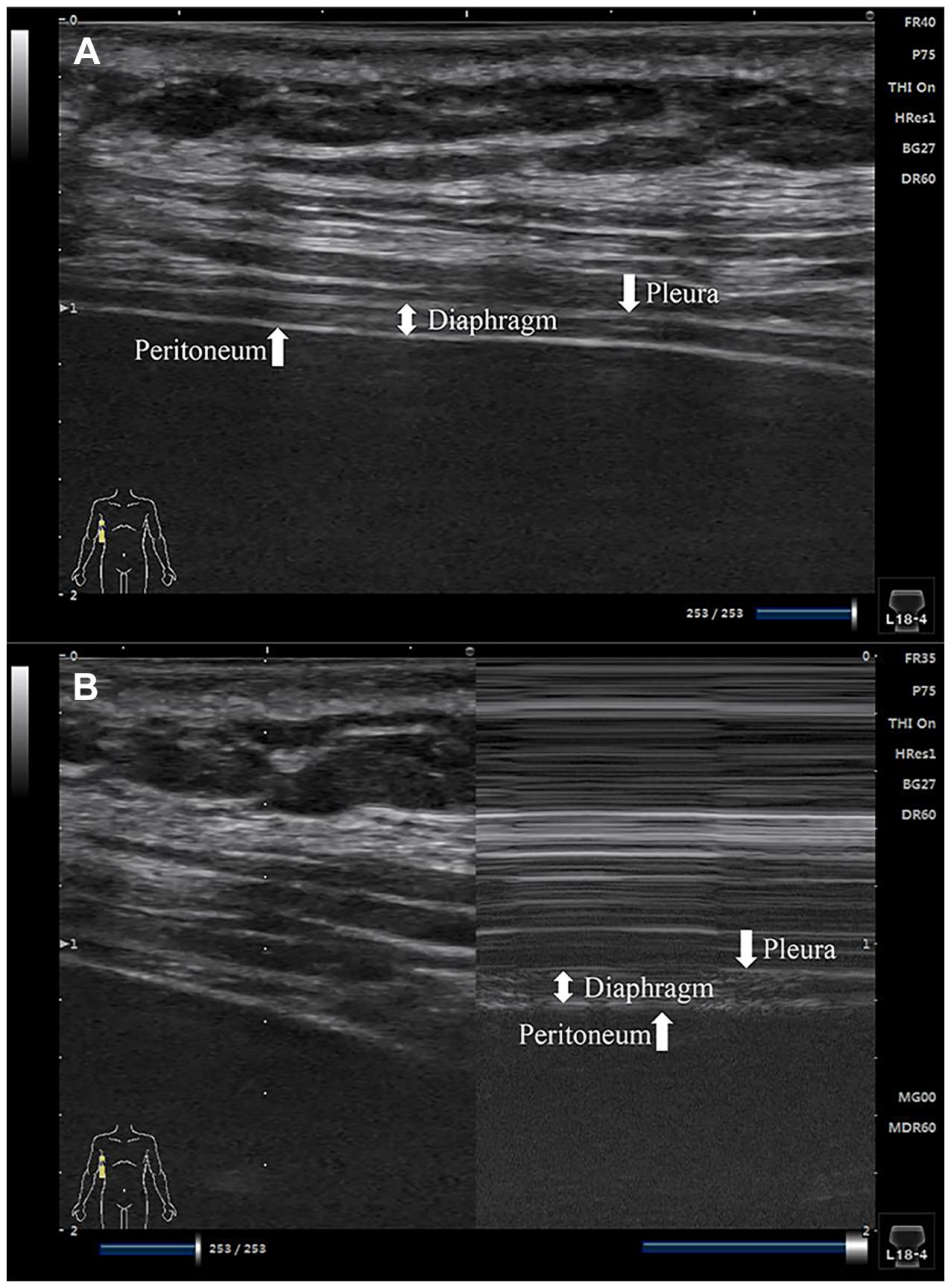
Images of the diaphragm ultrasound. **(A)** 2D mode diaphragm image; **(B)** M-mode diaphragm image.

The diaphragm’s thickness was assessed pre- and post-operatively using B-mode ultrasound. A linear array ultrasound probe, with a frequency range of 5–12 MHz, was positioned in the diaphragmatic zone of apposition - between the eighth and tenth costal spaces along the mid-axillary line. This specific zone allows for accurate measurement of changes in diaphragm thickness as it undergoes contraction and relaxation during respiratory cycles. In 2D mode, three parallel tissue layers - the pleura, diaphragm, and peritoneum - were visualized at a distance of 1.5–3 cm from the skin (refer to Figure 2A). Subsequently, conversion to M-mode was conducted with patients instructed to breathe calmly, enabling the capture of frozen screen images to monitor changes in diaphragm thickness (refer to Figure 2B).

During each assessment, three distinct sites within the zone of apposition were measured continuously over three respiratory cycles. The diaphragmatic thickness was calculated by averaging nine measurements taken. To evaluate the extent of diaphragmatic thickness change and subsequent recovery, two metrics were utilized. The first metric, thickening fractioning (TF), was computed as the percentage difference between thickness at the end of inspiration (TEI) and thickness at the end of expiration (TEE) [TF = (TEI - TEE) / TEE × 100%]. A TF ratio of 0.36 or lower suggests PORC. The second metric, diaphragmatic thickness recovery fractioning (TRF), was determined by comparing baseline values at the end of preanesthetic inspiration with values at the end of postoperative inspiration [TRF = (preanesthetic TEI - postoperative TEI) / preanesthetic TEI × 100%]. A non-positive TRF value indicates near-complete restoration of diaphragmatic function. Ultrasonography was performed by two skilled physicians who had undergone standardized training.

### Anesthesia procedure

Upon the patient’s arrival in the operating room, peripheral vein access was established in the forearm. Prior to anesthesia induction, various monitoring procedures were initiated, including non-invasive blood pressure measurement, electrocardiography, pulse oximetry, capnography, body temperature monitoring, and BIS (bispectral index) monitoring. Anesthesia was induced intravenously with 2.5 mg/kg of propofol and 0.5 μg/kg of sufentanil. The neuromuscular monitor was calibrated once the BIS value dropped below 60, followed by the administration of 0.6 mg/kg of rocuronium via intravenous injection for muscle relaxation. Subsequently, endotracheal intubation was performed, and anesthesia was maintained with propofol at a rate of 100–150 μg/kg/min and remifentanil at a rate of 0.3–0.5 μg/kg/min. Continuous rocuronium infusion was initiated at a rate of 0.3–0.4 mg/kg/h, with adjustments made in increments of 5–10 mg/h. During mechanical ventilation in volume-controlled mode, all participants were ventilated with a tidal volume of 8–10 mL/kg and an appropriate respiratory rate to ensure end-tidal carbon dioxide levels remained between 35 and 40 mmHg. Anesthetic infusions were adjusted as necessary by the anesthetist to maintain intraoperative BIS levels between 40–60 and PTC scores at 1–2. Following the completion of the procedure, administration of anesthetic agents was discontinued, and the patient was transferred to the post-anesthesia care unit (PACU) while still intubated. Once the patient achieved a TOF count of ≥2, a single dose of 2 mg/kg sugammadex was administered to reverse neuromuscular blockade. Extubation occurred after the patient regained consciousness, spontaneous breathing, and demonstrated responsiveness by opening their eyes, shaking hands, and maintaining head elevation for more than 5 s.

### Outcome variables

The study’s primary outcome variables were TF and TRF, determined by assessing diaphragmatic thickness at 0, 10, and 30 min, as well as 2 h post-extubation. Secondary outcome measures encompassed the duration from sugammadex administration to tracheal extubation, the length of stay in the PACU, and any hemodynamic alterations subsequent to sugammadex administration.

### Grouping and blind method

This study is a non-randomized controlled trial in which a dedicated research coordinator, not involved in the follow-up study, will conduct a review of the electronic medical record system. The coordinator will categorize patients into two groups (A and B) based on preoperative liver function test results and Child-Pugh grade standards. Patients will then be sequentially assigned identification numbers by the researchers.

To ensure blinding of the researchers, a designated study coordinator will oversee drug storage and preparation, as well as facilitate information exchange among the research team. Drug administration and recording of basic participant information will be carried out by a designated nurse. Another researcher will be responsible for patient follow-up and data collection from diaphragm ultrasound monitoring. Throughout the study duration, these research personnel will maintain separate records without access to each other’s data. For patient blinding, standardized appearance syringes and microsyringe pumps will be uniformly utilized for all patients during the procedure. Additionally, the same ultrasound machine will be used for diaphragm ultrasound monitoring across all participants in the study.

### Statistical analysis

IBM SPSS Statistics (IBM Corp, Version 26.0, Armonk, NY, 1989–2019) was utilized for statistical compilation and analysis. Categorical data were presented as frequencies and compared using either the χ^2^ test or Fisher’s exact test. Meanwhile, continuous data were expressed as mean ± standard deviation (SD) and compared between two groups using the independent samples t-test. A *p*-value of less than 0.05 was considered statistically significant.

For the Fisher exact test, it was determined that a sample size of 33 patients per group was required to achieve a test power of 80% with a type I error rate of 5%. The study aimed to detect PORC rates at 10 min post-extubation of 15% in the Child-Pugh A group and 46% in the Child-Pugh B group. Additionally, a total of 74 patients were planned for enrollment following a 1:1 ratio to account for a potential dropout rate of 10%.

## Results

The study cohort comprised 69 patients who underwent laparoscopic radical resection for hepatic tumors. Of these, two patients necessitated conversion to open liver resection, and one patient was transferred to the intensive care unit post-operation. Consequently, the final analysis encompassed 66 patients: 33 categorized as Child-Pugh A group and an additional 33 classified as Child-Pugh B group, enabling a comparative assessment.

### Baseline and surgical characteristics

The patients’ characteristics were equally distributed in both the groups as shown in Table 1, including age, gender, BMI, and comorbidities. Furthermore, the duration of surgery and anesthesia shows no significant differences between the two groups. Equivalent doses of anesthetics or sugammadex are administered during surgery for both groups.

### Outcome variables

**Figure 3:**
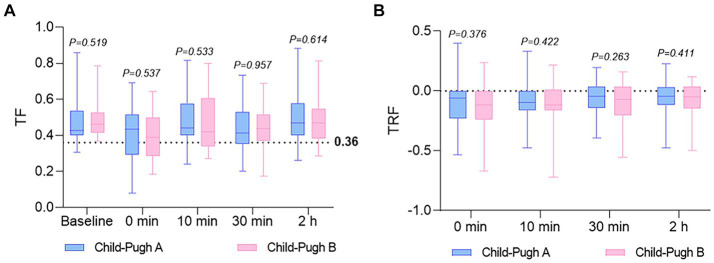
The distribution of TF and TRF values at various time points for both groups. **(A)** TF was calculated before anesthesia (baseline), and at 0 (extubation), 10, and 30 min, as well as 2 h in both groups. A TF ratio of 0.36 or lower suggests PORC. **(B)** TRF was calculated at 0 (extubation), 10, and 30 min, as well as 2 h in both groups. A non-positive TRF value indicates near-complete restoration of diaphragmatic function. Values are reported as median, upper and lower quartile. TF, thickening fractioning; TRF, thickness recovery fractioning.

Figure 3 displays the distribution of TF and TRF values at various time points for the two groups. No significant association between lack of recovery (TF ≤ 0.36 or TRF > 0) and Child-Pugh grades was observed. Nevertheless, a closer examination of Table 2 reveals that, in comparison to the Child-Pugh A group, patients in the Child-Pugh B group generally experience a decrease in average TF and TRF levels at 0, 10, and 30 min, as well as 2 h post-extubation. Furthermore, the analysis of Table 2 suggests a potential inclination for Child-Pugh B patients to have a higher risk of PORC (TF ≤ 0.36) immediately after extubation (at times 0 and 10), although this finding also lacks statistical significance.

**Table 2:** Primary outcome variables of the patients included TF values ≤ 0.36 and TRF values ≤ 0 in both groups at 0 (extubation), 10, and 30 minutes, as well as 2 hours after extubation.

		Child-Pugh A (*n* = 33)	Child-Pugh B (*n* = 33)	P
Patients with TF* ≤ 0.36 (PORC*)				
(1) 0 min	n (%)	12 (40)	15 (50)	0.453
	Mean (SD)	0.413 (0.152)	0.392 (0.121)	0.537
(2) 10 min	n (%)	5 (20)	10 (30)	0.142
	Mean (SD)	0.483 (0.146)	0.461 (0.142)	0.533
(3) 30 min	n (%)	8 (20)	7 (20)	0.769
	Mean (SD)	0.442 (0.131)	0.440 (0.120)	0.957
(4) 2 h	n (%)	5 (20)	6 (20)	0.741
	Mean (SD)	0.495 (0.133)	0.480 (0.120)	0.061
Patients with TRF* ≤ 0				
(1) 0 min	n (%)	27 (80)	25 (80)	0.547
	Mean (SD)	−0.094 (0.187)	−0.139 (0.218)	0.376
(2) 10 min	n (%)	28 (90)	25 (80)	0.353
	Mean (SD)	−0.085 (0.160)	−0.120 (0.188)	0.422
(3) 30 min	n (%)	22 (70)	24 (70)	0.592
	Mean (SD)	−0.056 (0.133)	−0.098(0.163)	0.263
(4) 2 h	n (%)	24 (70)	23 (70)	0.786
	Mean (SD)	−0.052 (0.143)	−0.080 (0.135)	0.411

*PORC, postoperative residual curarization; TF, thickening fractioning; TRF, thickness recovery fractioning.

**Figure 4:**
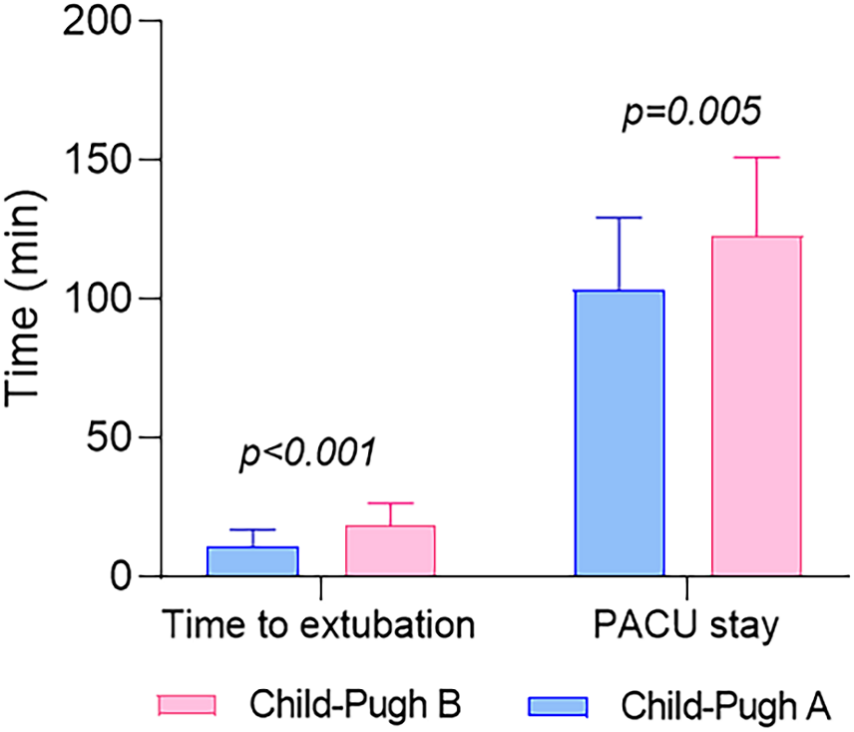
The distribution of the duration from sugammadex administration to tracheal extubation and the length of stay in the PACU values for both groups. Values are reported as mean and standard deviation.

**Figure 5:**
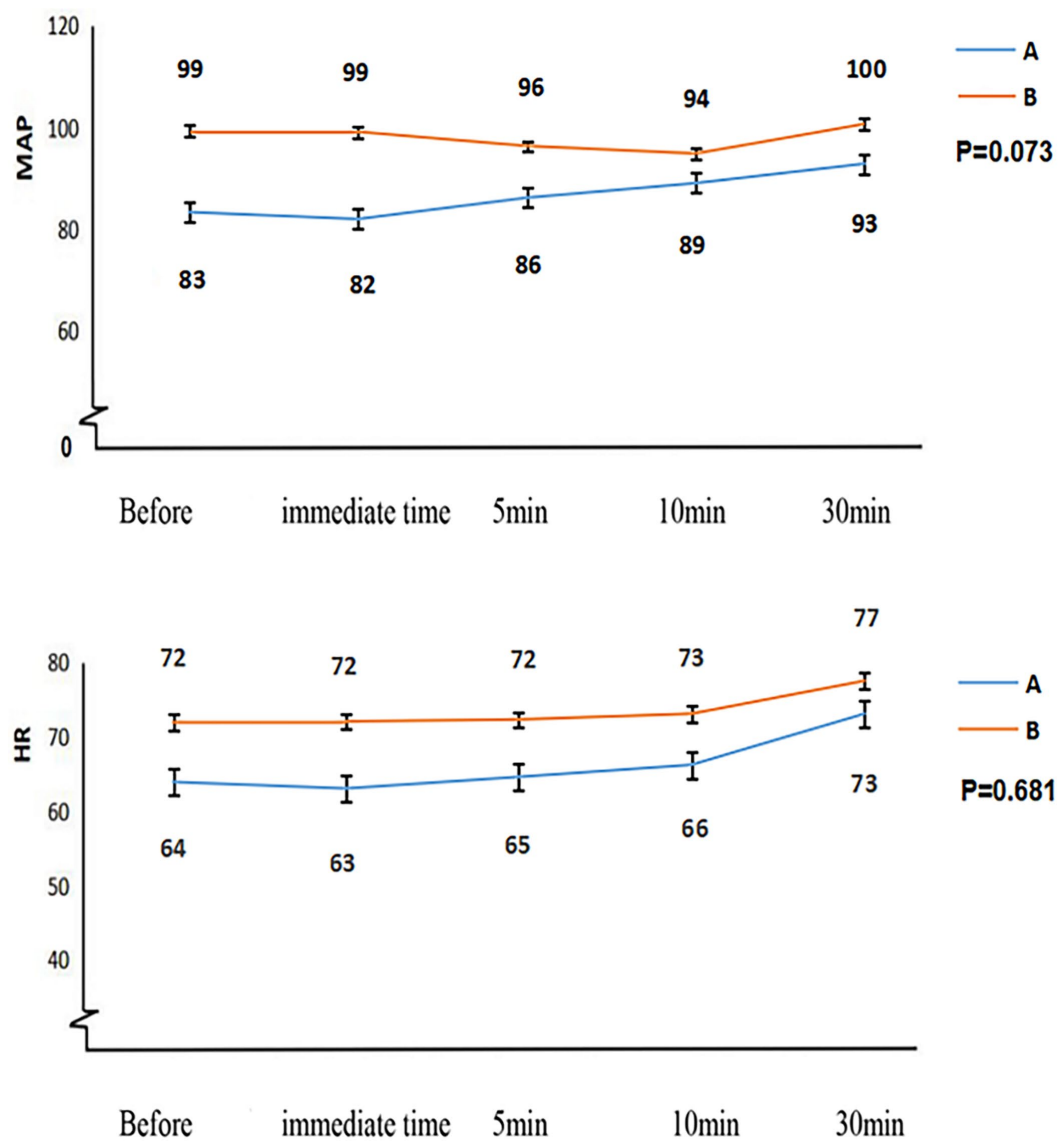
The hemodynamic changes in patients from both groups following the administration of sugammadex for rocuronium antagonism. HR, heart rate; MAP, mean arterial pressure.

Patients classified as Child-Pugh A experienced a significantly shorter duration from sugammadex administration to tracheal extubation (11 ± 6.1 min vs. 19 ± 8.0 min, *p* < 0.001) and a reduced length of stay in the PACU (103 ± 26.0 min vs. 123 ± 28.3 min, *p* = 0.005) than patients classified as Child-Pugh B, as outlined in Table 3 and Figure 4. Figure 5 demonstrates no noticeable differences in heart rate and mean arterial pressure following sugammadex administration.

**Table 3:** Secondary outcome variables of the patients s included the duration from sugammadex administration to tracheal extubation and the length of stay in the PACU in both groups.

	Child-Pugh A (*n* = 33)	Child-Pugh B (*n* = 33)	*p*
Duration from sugammadex administration to tracheal extubation (min; Mean, SD)	11(6.1)	19(8.0)	<0.001
Length of stay in the PACU* (min; Mean, SD)	103(26.0)	123(28.3)	0.005

*PACU, postanesthesia care unit.

## Discussion

The pharmacokinetics of rocuronium are notably variable in patients with liver disease, as indicated by reduced clearance and prolonged recovery time observed by some researchers (4, 5, 17). Consequently, liver dysfunction or hepatic surgery is considered one of the risk factors for a residual muscle-relaxant effect. Despite this, study has demonstrated that sugammadex can rapidly reverse neuromuscular blockade induced by continuous rocuronium infusion in patients with liver dysfunction and control groups undergoing hepatic surgery (18). Our study offers the assessment of sugammadex’s antagonistic impact on rocuronium in patients preoperatively categorized as Child-Pugh grades A and B, employing diaphragmatic ultrasound. The main finding, assessed through TF and TRF, reveals no significant difference between the two groups at each time point, although patients in the Child-Pugh B group generally exhibit an increase in PORC (TF ≤ 0.36) compared to the Child-Pugh A group at 0 and 10 min (Table 2). This observed trend may find support in the mean TF and TRF values for both groups across different time intervals (as illustrated in Table 2; Figure 3). These results suggest that the preoperative liver dysfunction of varying degrees could not significantly influence the reversal effect of sugammadex on rocuronium.

Neuromuscular monitors are commonly utilized intraoperatively to assess muscular blockade, but their use is restricted to unconscious patients due to the discomfort they may cause. The introduction of ultrasound has enabled noninvasive and bedside assessment of the function of the primary respiratory muscle, the diaphragm. Evaluation of postoperative paralysis has emerged as a focal point in clinical research within the fields of anesthesia and intensive care in this domain (19–21). Herein, we failed to show significant differences in TF and TRF between the two groups at each time point, which was the primary endpoint of our investigation (Table 2; Figure 3). However, it is not easy to differentiate whether the lack of a discernible difference between the two groups is genuine or attributable to limitations of the technique employed, wherein TF depends on patients’ characteristics such as height, weight, BMI, lung volume and sedation (13). Consequently, further studies under similar conditions are required.

With sugammadex, it is not always possible to definitively rule out PORC, particularly in cases involving the administration of low-dose sugammadex or the maintenance of deep neuromuscular blockade during the operation (22, 23). Despite promptly administering the recommended dose of sugammadex postoperatively in our study, a notable proportion of patients in both groups still encountered instances of PORC. As a result, anesthesiologists should remain highly vigilant for the occurrence of PORC in patients with impaired liver function, even when sugammadex has been used to antagonize rocuronium.

In a study by Fujita A et al., it was reported that the time from sugammadex administration to reaching a TOF ratio of 0.9 did not show a significant difference between the liver dysfunction group and the control group. The mean time in the liver dysfunction group was slightly longer than in the control group (2.2 min vs. 2.0 min, *p* = 0.44) (18). In a randomized controlled study conducted by Abdulatif M et al., it was found that sugammadex can rapidly antagonize moderate residual rocuronium-induced neuromuscular block in patients with Child class “A” liver cirrhosis undergoing liver resection, similar to the control group. And the times to achieve a TOF ratio of 0.9 were 3.1 min and 2.6 min after sugammadex administration in patients with liver cirrhosis and controls, respectively (*p* = 1.00) (24). However, in our present study, the mean time from sugammadex administration to tracheal extubation was 11 min in the Child-Pugh A group and 19 min in the Child-Pugh B group, with a significant difference observed between the two groups (*p* < 0.001; Table 3; Figure 4). This finding suggests a delayed recovery time in patients with more severe liver dysfunction.

Additionally, the mean time in our study population (11 min and 19 min) was notably prolonged compared to patients with liver dysfunction in the referenced study. The apparent discrepancy could be attributed primarily to the varying definitions of recovery criteria. Our study defines the time threshold based on subjective extubation standards, where extubation is considered after the patient has regained consciousness, initiated spontaneous breathing, and exhibited responsiveness by opening their eyes, shaking hands, and sustaining head elevation for over 5 s. In contrast, the previous research defines the time threshold based on established standards (TOF ratio to 0.9). Our choice not to employ neuromuscular monitors in assessing TOF ratios in extubated patients aims to prevent discomfort that conscious individuals might encounter. The delay noted could be linked to the pharmacokinetics of intravenous propofol in patients with liver disease. Additionally, aligning our results with those of Deana et al. (25), it is essential to highlight that recovery time with sugammadex in hepatopathic patients may significantly surpass that in other surgical settings, a factor that should be taken into consideration in clinical practice. A population pharmacokinetic-pharmacodynamic interaction model of sugammadex has been employed to simulate the reversal of rocuronium-induced neuromuscular blockade in patients with hepatic impairment. The model suggests a potential prolongation of recovery time after the administration of sugammadex in patients with hepatic impairment compared to that in healthy subjects (26).

In this study, another endpoint used to assess recovery time is the duration of stay in the PACU. Patients classified as Child-Pugh A had a PACU stay of 103 min, which was significantly shorter than those categorized as Child-Pugh B, with a stay of 123 min (*p* = 0.005; Table 3; Figure 4). This observation implies that patients with poorer hepatic function may experience delayed postoperative recovery. However, this finding appears contradictory to previous studies, where there was no significant difference in PACU stay duration between cirrhotic patients (Child-Pugh A) and the control group following sugammadex administration. Specifically, the PACU stay durations were 23.0 min for the patient group and 22.8 min for the control group, respectively (24). Moreover, the duration of stay in the PACU in our study is notably longer than that reported in the aforementioned study. The fact that our patients received continuous deep neuromuscular blockade throughout the surgical procedure could potentially explain this difference.

The outstanding tolerability and exceptional safety profile of sugammadex have been consistently demonstrated in numerous studies (18, 27–30). Nonetheless, there has been limited clinical research investigating the safety and effectiveness of sugammadex in reversing deep residual neuromuscular blockade induced by rocuronium in patients with liver dysfunction. Our investigation uncovered no instances of adverse events associated with the administration of sugammadex. Furthermore, we observed minimal impact on hemodynamic stability in patients with impaired liver function (Figure 5).

Our study has several limitations. Firstly, we focused exclusively on patients with Child-Pugh grades A and B due to the limited number of eligible patients with Child-Pugh grade C. Consequently, our findings may not be generalizable to patients with Child-Pugh grade C. Secondly, the current accepted standard for diagnosing PORC in clinical trials is objective neuromuscular monitoring. Thirdly, the diaphragm, being a thin and flat muscle, exhibits thickness fluctuations ranging from 0.30 cm to 0.34 cm during respiratory cycles (31). Therefore, the accuracy of our data relies on the proficiency of ultrasonographic measurements of the diaphragm. However, all ultrasonographic measurements in our study were conducted by a well-trained investigator with extensive experience. Consequently, further investigation is warranted to examine the correlation between perioperative liver dysfunction and the effect of sugammadex on rocuronium using diaphragm ultrasound.

## Conclusion

Given the results obtained through diaphragm ultrasonography in this study, there may not be a significant correlation between preoperative Child-Pugh grade and the effectiveness of sugammadex in reversing rocuronium. Further research is essential to clarify the clinical utility of diaphragmatic ultrasonography for evaluating the effect of sugammadex on rocuronium in patients with liver dysfunction.

## Data availability statement

The raw data supporting the conclusions of this article will be made available by the authors, without undue reservation.

## Ethics statement

The studies involving humans were approved by Medical Ethics Committee, Union Hospital, Tongji Medical College, Huazhong University of Science and Technology, China. The studies were conducted in accordance with the local legislation and institutional requirements. The participants provided their written informed consent to participate in this study. Written informed consent was obtained from the individual(s) for the publication of any potentially identifiable images or data included in this article.

## Author contributions

YS: Writing – original draft, Validation. SS: Funding acquisition, Methodology, Writing – original draft. RC: Project administration, Software, Writing – review & editing. JS: Investigation, Writing – review & editing. XC: Conceptualization, Supervision, Writing – review & editing. YL: Conceptualization, Supervision, Writing – original draft. SY: Conceptualization, Methodology, Visualization, Writing – review & editing.
